# Contraceptive implant removal: Case report and the role of an ultrasound-trained anesthesiologist in perioperative localization and regional anesthesia

**DOI:** 10.1016/j.jatmed.2025.11.003

**Published:** 2025-12-16

**Authors:** Austin T. Nguyen, Matthew W. Swisher

**Affiliations:** a School of Medicine, University of California San Diego, La Jolla, CA 92093, USA; b Department of Anesthesiology, University of California San Diego, San Diego, CA 92093, USA

**Keywords:** Ultrasound guidance, Peripheral nerve blocks, Contraceptive implant, Regional anesthesia

## Abstract

**Background:**

Contraceptive implants offer a long-term reversible option for contraception. While palpable implants can be removed under local anesthesia in an office setting, nonpalpable implants can present greater safety challenges and may increase the overall complication rate of removal.

**Case presentation:**

We present the case of a 28 year-old female who presented for an elective upper extremity contraceptive implant removal. The implant could not be palpated, and imaging confirmed subfascial placement of the implant in muscle. Preoperatively, her implant was identified on ultrasound within the biceps brachii muscle and mapped to guide the dissection. An ultrasound-guided nerve block of the intercostobrachial and medial brachial cutaneous nerves was performed with subfascial deposition of local anesthetic. The implant was then successfully removed without any additional local anesthesia or analgesia.

**Conclusions:**

This case report highlights the role of anesthesiologist-directed localization and ultrasound-guided nerve blocks.

## Background

Contraceptive implants offer a long-term reversible option for contraception. These implants contain hormonal contraceptive agents (etonogestrel or levonorgestrel) and are designed to be placed subdermally, usually in the medial upper arm of the non-dominant extremity, for 3–5 years depending on the implant.^1^, ^2^ During the office-based insertion procedure, an applicator or trocar is inserted into the desired implant area under local anesthesia, and the implant is deployed. Once the duration of the implant is exceeded (or fertility/removal is desired), palpable implants are then removed under local anesthesia in the office setting. While palpable implants can be removed under local anesthesia in an office setting, nonpalpable implants can present greater safety challenges and may increase the overall complication rate of removal.^3^

If the implant is not palpable, the general guideline is to use ultrasound to confirm the implant’s presence and position. With ultrasound assistance, these nonpalpable contraceptive implants can be localized and removed by specialized referral centers in either the office setting or in the operating room depending on the depth and surrounding anatomy. Vascular and nerve injuries have been described as a result of the more complicated extraction procedure of nonpalpable implants.^3^ While the use of ultrasound localization of these deeper implants has been well described, there have been no case reports discussing the use of regional anesthesia to assist with the removal procedure.

## Case presentation

A 28-year-old female with a history of depression, posttraumatic stress disorder, and chronic low back pain presented for an elective left upper extremity contraceptive implant removal. Her contraceptive implant (etonogestrel implant, NEXPLANON®, Merck & Co, Inc, Rahway, NJ, USA) was originally placed in an outpatient clinic in July of 2017. According to the report, the initial device placement was uncomplicated and and performed in accordance with manufacturer recommendations, using the provided applicator in a subdermal fashion along the upper left medial arm. Subsequent, the patient reported intermittent “pinching” pain in her left arm, thus necessitating desire for removal. Outpatient radiographic imaging localized the implant in her left medial upper arm, and ultrasound imaging seemed to indicate subfascial placement in her left biceps brachii muscle 8 mm from the skin surface. She was then evaluated by orthopaedic surgery for operative removal of her implant and scheduled for outpatient surgery.

**Fig. 1:**
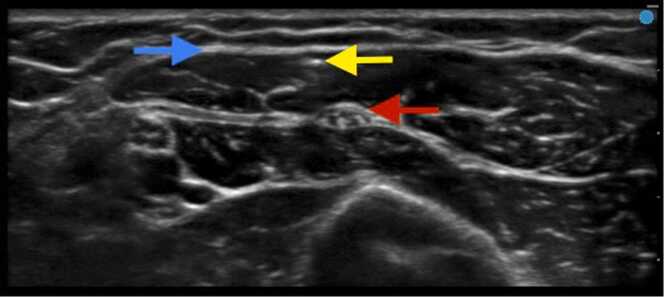
Cross-section ultrasound image of the hyperechoic implant (yellow arrow) within the biceps brachii muscle (fascia depicted as blue arrow) in close proximity to the musculocutaneous nerve (red arrow) and axillary sheath. Depth markers are seen on the right side of the image (total depth of the ultrasound image is approximately 2.3 cm).

Upon presentation on the day of surgery, the implant could not be palpated by the surgeon and a bedside ultrasound examination was requested to assist with localization. An ultrasound examination with a high-frequency linear probe (15–6 MHz, FUJIFILM SonoSite, Inc, Bothell, WA, USA) was performed in the preoperative area by an anesthesiologist fellowship-trained in ultrasound-guided regional anesthesia. It revealed that the implant was located in the biceps brachii muscle 2 mm deep to the biceps brachii fascia in relatively close proximity to the axillary sheath superficial to the musculocutaneous nerve (Fig. 1). Thus, the skin overlying the implant was marked using ultrasound to guide the incision and operative dissection. Of note, the implant had migrated proximally and was remote (>4 cm) from the previous punctate skin scar from the insertion procedure.

**Fig. 2:**
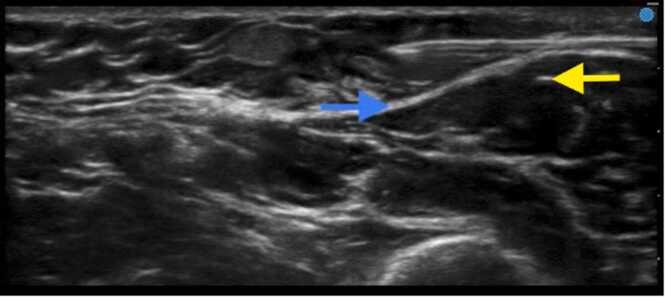
Ultrasound-guided intercostobrachial and medial brachial cutaneous nerve blocks. Fascia overlying the biceps brachii muscle depicted as a blue arrow and implant as a yellow arrow. Depth markers are seen on the right side of the image (total depth of the ultrasound image is approximately 2.3 cm).

To provide anesthesia and analgesia for the operation, ultrasound-guided nerve blocks targeting the intercostobrachial and medial brachial cutaneous nerves were performed, given the expected innervation to that area of the upper arm. A 22-gauge needle was inserted superficial to the biceps brachii fascia and axillary sheath under direct ultrasound guidance via an in-plane transverse approach approximately 2 cm proximal to the proximal end of the implant (Fig. 2). A total of 10 mL of 2 % lidocaine with 1:400,000 of epinephrine was injected to anesthetize both nerves after negative aspiration. This volume was chosen to ensure surgical anesthesia along the length of anticipated incision. Subsequently, in order to fully anesthetize the surgical dissection into the biceps brachii fascia and muscle, a subfascial injection of 5 mL of the same injectate was performed just proximal to the implant in a similar ultrasound-guided in-plane fashion.

**Fig. 3:**
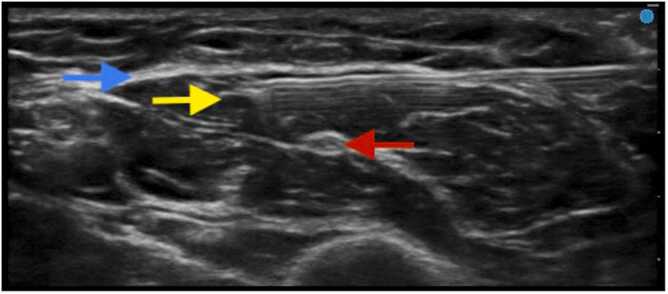
Ultrasound-guided deposition of local anesthetic deep to biceps brachii fascia (fascia labeled with blue arrow, implant with yellow arrow, and musculocutaneous nerve as red arrow). Depth markers are seen on the right side of the image (total depth of the ultrasound image is approximately 2.3 cm).

After confirmation of adequate superficial anesthesia of the medial upper arm, the patient was taken into the operating room for surgery. Surgery was then performed uneventfully under moderate sedation. A longitudinal incision was made overlying the preoperative ultrasound-guided mapping, and dissection was carried down through the subcutaneous tissue into the biceps brachii fascia and muscle (Fig. 3). The implant was then found just deep to the fascia and was subsequently removed from the muscle (Fig. 3). Of note, the patient did not require any additional analgesics or intraoperative local anesthetic infiltration. Her pain scores in the recovery unit were zero out of ten throughout her recovery. She was subsequently discharged from the recovery unit to home, and her nerve blocks provided full analgesia until the blocks wore off after 6 h. No adverse events or side effects from the nerve blocks were observed.

## Discussion

This case report illustrates the expanding role of anesthesiologists in the application of point-of-care ultrasound and regional anesthesia to localize soft tissue implants, guide surgical dissection, and provide anesthesia and analgesia with ultrasound-guided nerve blocks. While ultrasound localization of contraceptive implants has been extensively described, the potential role of anesthesiologists trained in ultrasound has not. The use of preoperative ultrasound for mapping nonpalpable implants on the day of surgery reduces the time, dissection, and tissue trauma required to locate the implant. Other imaging modalities such as computed tomography and magnetic resonance imaging are less helpful with real-time localization on the day of surgery given their constraints. While plain radiography and fluoroscopy can be of assistance preoperatively and intraoperatively, these modalities are limited in comparison to ultrasound imaging mainly for depth of insertion, which is critical for appropriate localization and dissection. In addition, ultrasound-guided nerve blocks of the intercostobrachial and medial brachial cutaneous nerves for surgical anesthesia for these implant removals in the medial upper arm have not been described and can minimize pain and trauma during the case.

In our case, the implant migrated quite proximally from the original insertion site, and preoperative ultrasound mapping allowed the surgeon to minimize the planned incision, guide surgical dissection, and prevent trauma to nearby structures. There have been multiple case reports involving neurovascular injuries during contraceptive implant removals which have resulted in significant morbidity.^4^, ^5^, ^6^ While the etiology of these injuries is not always known, surgical dissection and inadvertent trauma to nearby nerves and vasculature likely plays a significant role since these implants can be challenging to locate in the operating room and their proximity to upper extremity nerves and vasculature. Given the proximity of the axillary sheath, vessels, and nerves to the location of these implants (medial upper arm), injuries may occur from initial insertion or attempts to remove deeply placed implants.

In addition to localization and mapping on the day of surgery, these cases are well suited for regional anesthesia. Interestingly, ultrasound-guided nerve blocks have not been described previously for contraceptive implant removal surgeries. After localization of the implant and mapping, ultrasound-guided intercostobrachial and medial brachial cutaneous nerve blocks can be easily performed for the subdermal dissection down to the implant. In addition, as many nonpalpable implants are deeper than subdermal (as in subfascial in our case), the entire dissection plane can be anesthetized with ultrasound guidance. This approach avoids the need for general anesthesia and additional local anesthetic infiltration in the operating room, which may distort the operative anatomy near terminal nerves and vasculature. In our case, the implant was easily visualized just deep to the biceps brachii fascia and within the biceps brachii muscle. Further ultrasound-guided subfascial deposition of local anesthetic with our block needle provided surgical anesthesia for the operative removal.

As a single case, there are numerous limitations inherent to our technique. Given the lack of comparative data, it remains unknown whether this is the optimal approach for localizing and anesthetizing nonpalpable contraceptive implants. In addition, no long-term follow-up data were obtained. While these techniques could be applicable to locating other types of implantable devices and foreign bodies, those particular scenarios remains to be investigated. Future directions should include the investigation of this technique in a prospective fashion in addition to studying ultrasound localization and regional anesthesia for other implantable devices and foreign bodies.

## Conclusions

This case report illustrates the valuable role of anesthesiologists in managing complex contraceptive implant removals using point-of-care ultrasound and ultrasound-guided nerve blocks. The described case, involving a contraceptive implant that migrated from its initial insertion site, highlights the need for specialized techniques and coordination between anesthesiologists and surgeons to ensure safe and effective removal. Ultrasound localization enabled accurate identification of the implant’s position in relation to sensitive structures like the axillary sheath and musculocutaneous nerve, while ultrasound-guided nerve blocks provided surgical anesthesia and minimized the risk of surgical dissection and trauma. This approach may prevent complications, enhance patient outcomes, and expand the role of ultrasound-trained anesthesiologists in complex implant removal cases and possibly other soft tissue foreign body removal surgeries.

## CRediT authorship contribution statement

**Matthew W. Swisher:** Conceptualization, Data curation, Investigation, Methodology, Project administration, Supervision, Validation, Visualization, Writing – original draft. **Austin T. Nguyen:** Investigation, Methodology, Validation, Visualization, Writing – original draft. All authors have read and agreed to the published version of the manuscript.

## Consent for publication

Written informed consent was obtained from the patient for the procedure and written Health Insurance Portability and Accountability Act (HIPAA) authorization was obtained from the patient for the publication of this case report.

## Ethical statement

The university’s Institutional Review Board (University of California San Diego, San Diego, CA, USA) waives any review requirements for case reports. This manuscript adheres to the applicable EQUATOR guideline.

## Funding

This research received no external funding.

## Declaration of competing interest

The authors declare the following financial interests/personal relationships which may be considered as potential competing interests: Name: Matthew W. Swisher, MD, MS. Conflicts of Interest/Financial Disclosures: The University of California San Diego has received funding and/or product from the following companies for other research studies of this author: Epimed International (Farmers Branch, TX, USA), SPR Therapeutics (Cleveland, OH, USA), Infutronix (Natick, MA, USA), and Avanos Medical (Irvine, CA, USA). This author is a paid consultant for Vertex Pharmaceuticals (Boston, MA, USA).

## Data Availability

All data are included in the article.

## References

[1] Hohmann H., Creinin M.D. (2007). The contraceptive implant. Clin Obstet Gynecol.

[2] McNicholas C., Swor E., Wan L. (2017). Prolonged use of the etonogestrel implant and levonorgestrel intrauterine device: 2 years beyond Food and Drug Administration-approved duration. Am J Obstet Gynecol.

[3] Soler-Perromat J.C., Isern-Kebschull J., Del Amo M. (2024). Ultrasound-guided minimally invasive removal of deep contraceptive implants: outcomes and challenges. Quant Imaging Med Surg.

[4] Monteiro R.B., Metzger P.B., de (2020). Traumatic pseudoaneurysm in brachial artery after removal of a subdermal contraceptive implant. J Vasc Bras.

[5] Lefebvre R., Hom M., Leland H. (2018). Peripheral nerve injury with Nexplanon removal: case report and review of the literature. Contracept Reprod Med.

[6] Christensen J.M., Caggiano N.M., Giladi A.M. (2018). Median nerve injury after removal of subdermal implantable contraceptive. Hand.

